# In Vivo Analysis of Human Immune Responses in Immunodeficient Rats

**DOI:** 10.1097/TP.0000000000003047

**Published:** 2020-03-31

**Authors:** Séverine Ménoret, Laure-Hélène Ouisse, Laurent Tesson, Séverine Remy, Claire Usal, Aude Guiffes, Vanessa Chenouard, Pierre-Joseph Royer, Gwenaelle Evanno, Bernard Vanhove, Eliane Piaggio, Ignacio Anegon

**Affiliations:** 1 Centre de Recherche en Transplantation et Immunologie UMR1064, INSERM, Université de Nantes, Nantes, France.; 2 Institut de Transplantation Urologie Néphrologie (ITUN), CHU Nantes, Nantes, France.; 3 Nantes Université, CHU Nantes, Inserm, CNRS, SFR Santé, Inserm UMS 016, CNRS UMS 3556, Nantes, France.; 4 Xenothera, Nantes, France.; 5 PSL Research University, Institut Curie Research Center, INSERM U932, Paris, France.

## Abstract

**Background.:**

Humanized immune system immunodeficient mice have been extremely useful for the in vivo analyses of immune responses in a variety of models, including organ transplantation and graft versus host disease (GVHD) but they have limitations. Rat models are interesting complementary alternatives presenting advantages over mice, such as their size and their active complement compartment. Immunodeficient rats have been generated but human immune responses have not yet been described.

**Methods.:**

We generated immunodeficient Rat Rag−/− Gamma chain−/− human signal regulatory protein alpha-positive (RRGS) rats combining Rag1 and Il2rg deficiency with the expression of human signal regulatory protein alpha, a negative regulator of macrophage phagocytosis allowing repression of rat macrophages by human CD47-positive cells. We then immune humanized RRGS animals with human peripheral blood mononuclear cells (hPBMCs) to set up a human acute GVHD model. Treatment of GVHD was done with a new porcine antihuman lymphocyte serum active through complement-dependent cytotoxicity. We also established a tumor xenograft rejection model in these hPBMCs immune system RRGS animals by subcutaneous implantation of a human tumor cell line.

**Results.:**

RRGS animals receiving hPBMCs showed robust and reproducible reconstitution, mainly by T and B cells. A dose-dependent acute GVHD process was observed with progressive weight loss, tissue damage, and death censoring. Antihuman lymphocyte serum (L1S1) antibody completely prevented acute GVHD. In the human tumor xenograft model, detectable tumors were rejected upon hPBMCs injection.

**Conclusions.:**

hPBMC can be implanted in RRGS animals and elicit acute GVHD or rejection of human tumor cells and these are useful models to test new immunotherapies.

## Abstract 2

Supplemental Digital Content is available in the text.

## INTRODUCTION

Immunodeficient mice in particular have been extremely useful for the analysis of in vivo functions and biological performances of different molecules that are immunogenic, and additionally, to humanize different tissues to generate a variety of human pathophysiological models.^1,2^ Nevertheless, alternative models are needed because their small size is an obstacle for the development of different models. Furthermore, some inherent characteristics are also obstacles for other applications. For example, most mouse inbred strains show levels of complement much lower than those of rat and human sera.^3^ Rats are a useful alternative because they are 10-fold bigger than mice allowing more frequent blood sampling and in larger volumes, to harvest larger number of cells and to perform surgical procedures, such as implantation of cells into organs such as the brain, prostate or ovaries. Furthermore, in some models, rats have proven to better reproduce pathologies observed in humans, such as Duchenne disease following gene inactivation of dystrophin^4^ and in these models availability of immunodeficient rats would be very useful to test treatments such as human stem cells or gene therapy without the interference of immune responses. Finally, there are some immunological similarities between rats and humans that are not presented in mice. For example, activated T cells in humans and rats, but not in mice, express major histocompatibility complex class II and FoxP3 molecules and expression of CD8 and CD4 are detected on human and rat, but not mouse macrophages.^5^

Immunodeficient rats with single or combined deficiencies in genes involved in immune adaptive immune responses, such as *Prkdc, Rag1, Rag2*, and *Il2rg*, have been described.^6-10^ These rats, although capable of accepting certain human tissues, such as tumors and skin grafts, were refractory to humanization using isolated human cells, such as CD34+ hematopoietic precursors or peripheral blood mononuclear cells (PBMCs) and showed low efficacy of liver humanization with hepatocytes.^6,8,10^

In mice immunodeficient for the genes mentioned above, hematopoietic or liver humanization was also inefficient. These humanizations are prevented by a molecular incompatibility between mouse macrophage signal regulatory protein alpha (SIRPa) and human CD47 expressed on all hematopoietic cells, that normally provide “don’t eat me” signals. Humanization was greatly ameliorated in immunodeficient mice in which SIRPa/human CD47 interaction was restored.^1,2,11,12^ The finding that the nonobese diabetic mice (NOD) strain has a spontaneous mutation that allows mouse macrophage SIRPa and human CD47 interactions explained the better humanization obtained with this strain.^13^ However, the NOD strain has also genetic deficiencies in the complement system^14^ and lack natural killer (NK) cells.^15^ These are obstacles for its use in the evaluation of antibody effector functions such as complement-dependent cytotoxicity (CDC) and antibody-dependent cellular cytotoxicity (ADCC). Other mouse strains, such as C57/Bl6, were genetically modified to introduce human SIRPa (hSIRPa) in macrophages^11^ but nevertheless, all inbred mouse strains including C57/Bl6 show much lower levels of complement as compared with human or rat serum.^3^

A recent publication described immunodeficient rats expressing hSIRPa that were immune humanized but analyses of human immune responses were not reported.^16^ To obtain humanization of the immune system using PBMCs in rats and then analyze immune responses, we crossed rats that are deficient for *Rag1* and *Il2rg* (Rat Rag1-deficient, Il2rg-deficient [RRG] animals)^10^ with a transgenic rat line expressing human hSIRPa in rat macrophages^17^ to obtain RRGS animals. We show that RRGS animals present efficient, robust, and reproducible humanization of immune cells using PBMCs, with the development of acute graft versus host disease (aGVHD) if sufficient numbers of human PBMCs (hPBMCs) were injected. GVHD could be inhibited by treatment with a new antihuman lymphocyte polyclonal antibody produced in pigs, mainly functioning through complement-mediated cytotoxicity. We also describe an efficient human immune response against human tumor cells in PBMC-humanized RRGS animals. In summary, we established for the first time, a robust human immune response in immunodeficient rats.

## MATERIALS AND METHODS

### Animals

RRG generated using meganucleases and transcription activator-like effector nuclease^10^ and hSIRPa transgenic rats generated using a human bacterial artificial chromosome with promoter sequences^17^ have been previously described and were crossed to obtain RRGS rats that were maintained under specific pathogen-free conditions. Rats were genotyped using microcapillary electrophoresis as previously described.^18^ Wild type Sprague-Dawley (SD/Crl) rats were from Charles River (L’Arbresle, France). All animal care and procedures performed in this study were approved by the Animal Experimentation Ethics Committee of the Pays de la Loire region, France, in accordance with the guidelines from the French National Research Council for the Care and Use of Laboratory Animals (Permit Numbers: Apafis 692, Apafis 17305, and Apafis 17306). All efforts were made to minimize suffering. The rats were housed in a controlled environment (temperature 21°C ± 1°C, 12-hour light/dark cycle).

### Cytofluorimetry and Antibodies

Single-cell suspensions from the spleen, thymus, bone marrow, and lymph nodes (LN) were prepared as described previously.^19^ Cell suspensions were analyzed using fluorescein isothiocyanate (FITC)-conjugated mouse antirat CD3 (clone G4.18) and FITC-conjugated mouse antirat T cell receptorαβ. Allophycocyanin (APC)-conjugated mouse antirat IgD (clone MARD-3) was obtained from AbD Serotec (Oxford, United Kingdom). FITC-conjugated mouse antirat IgM μ chain was bought from Jackson ImmunoResearch Laboratories (West Grove, PA). APC-conjugated mouse antirat CD161 (clone 3.2.3), phycoerythrin (PE)-conjugated mouse antirat CD45R (rat B220; clone His 24), PE-conjugated mouse antirat CD4 (clone OX35), and APC-conjugated mouse antirat CD8 (clone OX8) were from AbD (Serotec), and FITC-conjugated mouse antirat CD172a (clone OX41). PE-conjugated antihuman SIRPA monoclonal antibody (clone REA144, Miltenyi), Recombinant human CD47-Fc (hCD47-Fc, R&D Systems), PE-Cy7-conjugated antihuman CD45 (clone H130, BD Biosciences, Franklin Lakes, NJ), PE-conjugated antihuman CD3 (clone HIT3a, BD Biosciences), APC-conjugated antihuman CD56 (clone HCD56, Biolegend), Pacific Blue-conjugated antihuman CD14 (clone M5E2, BD Biosciences), and FITC-conjugated antihuman CD19 (clone HIB19, BD Biosciences).

The incubation period was 30 minutes at 4°C, and the analysis was performed with a FACSVerse system (BD Biosciences) and FlowJo software (Tree Star, Ashland, OR).

### Immune Humanization Using Human PBMCs

hPBMCs from healthy volunteers were isolated from buffy coat preparations using Ficoll-Hypaque density gradients and freshly injected intravenously (iv) into 3- to 4-week-old RRG or RRGS animals (210–350 g). PBMCs were injected in different numbers depending on the model as indicated in the respective sections.

### Treatment With Liposomes Containing Clodronate

Clodronate liposomes were purchased from Liposoma B.V. (the Netherlands; www.clodronateliposomes.org) and prepared as recommended.^20^ Briefly, rats were weighed, and 10 mL/kg of suspended solution was administered intraperitoneally twice a week.

### Generation of a New Antihuman Lymphocyte Polyclonal Antibody Produced in Pigs

Low immunogenicity antilymphocyte serum (LIS1) is a swine polyclonal IgG antihuman T lymphocyte, devoid of specific sialic acid *N*-glycolylneuraminic acid and α1–3 galactose carbohydrate xenoantigens to reduce the potential for adverse events such as serum sickness disease and allergy. It was generated by Xenothera (Nantes, France) by immunization of genetically modified pigs deficient for CMP-*N*-acetylneuraminic acid hydroxylase and in α1–3 galactosyltransferase enzymes with cells derived from a human leukemia T-cell line. LIS1 was purified from serum by affinity and ion-exchange chromatography steps and formulated in physiological buffer containing polysorbate 80.

### In Vitro CDC Against hPBMCs With LIS1

hPBMCs were isolated from 3 healthy donors by Ficoll (Eurobio, France) density gradient centrifugation. 2.5 × 10^5^ PBMC were incubated with increasing doses of purified pig LIS1 IgG antibodies or control nonimmune (67p) IgG in phosphate buffer solution (PBS) 1% bovine serum albumin (BSA) (Merck, France) for 30 minutes at 4°C. PBMC were then washed twice with PBS 1% BSA and resuspended on ice in 25 µL of neat human, mouse or rat serum. After 30 minutes incubation at 37°C, PBMC were washed twice with PBS 1% BSA on ice and resuspended in 100 µL of propidium iodide at 4 µg/mL (Merck, France). Cell cytotoxicity was determined by the % of PI-positive cells on a Canto II flow cytometer (BD Biosciences).

#### aGVHD Model and Treatment With LIS1

Rats were injected iv with hPBMCs (430 × 10^6^ cells/kg). aGVHD clinical evaluation included weight loss, physical activity, hutch posture, skin lesions, diarrhea, and fur texture that generated a clinical score (for each no = 0; yes = 1). Analyses of alanine and aspartate transaminases (ALT and AST, respectively) were performed in sera using standard biochemical assays.

For evaluation of LIS1 activity, rats were injected iv with PBMCs (215 × 10^6^ cells/kg) and intraperitoneal with LIS1 or control nonimmune pig IgG (67p) at 40 mg/kg at day 0 and biweekly during 28 days.

### B2 Breast Cancer Cell Line

This cell line was generated from a fresh sample of a metastatic LN from a breast cancer patient without neoadjuvant chemotherapy, undergoing primary partial mastectomy with axillary LN dissection at Institut Curie Hospital (Paris, France) in accordance with institutional ethical guidelines. Clinicopathological characteristics of the tumor were invasive breast carcinoma of no specific type, estrogen receptor-positive, progesterone receptor-positive, HER2 negative, and grade 3 tumor with a Ki67 of 45%. The LN sample was cut into small fragments, digested with 0.1 mg/mL Liberase TL (Merck, France) in the presence of 0.1 mg/mL DNase (Merck, France) for 30 minutes in CO_2_ independent medium. Cells were filtered on a 40-μm cell strainer (BD Biosciences) and washed. Cells were stained with PERCP-e710 anti-EPCAM (Thermo Fisher Scientific, France) and APC-Cy7 anti-CD45 (BD) and Dapi. Tumor cells were isolated as EPCAM (+) CD45(−) by cell sorting on a FACSAria instrument (BD Biosciences). Cells were grown in RPMI 1640 10% bovine serum (Thermo Fisher Scientific, Waltham, MA) in a 48 well plate. Cells were characterized by ploidy evaluation by cytofluorometry analysis, comparative genomic hybridization, and immunochemistry.

### Immune Responses Against Human Tumor Cells

Human mammal cancer cells from line B2 (15 × 10^6^ cells) were injected subcutaneously in 12-week-old RRGS animals. hPBMCs (161 × 10^6^ cells/kg) were injected iv when tumors were palpable. Tumors were measured every 2–3 days (length [*a*] and width [*b*]) in millimeters using calipers, and tumor volumes (*V*) were calculated using the formula *V* = *ab*2/2, where *a* is the length of the 2 measurements.

### Statistical Analysis

Results are presented as means ± SD. Statistical analysis between samples was performed by a Mann-Whitney *U* test and for graft survival by a Kaplan-Meier test, using GraphPad Prism 4 software (GraphPad Software, San Diego, CA). Differences associated with probability values of ^a^*P* < 0.05, ^b^*P* < 0.005, ^c^*P* < 0.0002, and ^d^*P* < 0.0001 were considered statistically significant.

## RESULTS

### Generation and Characterization of RRGS Animals

**FIGURE 1.**
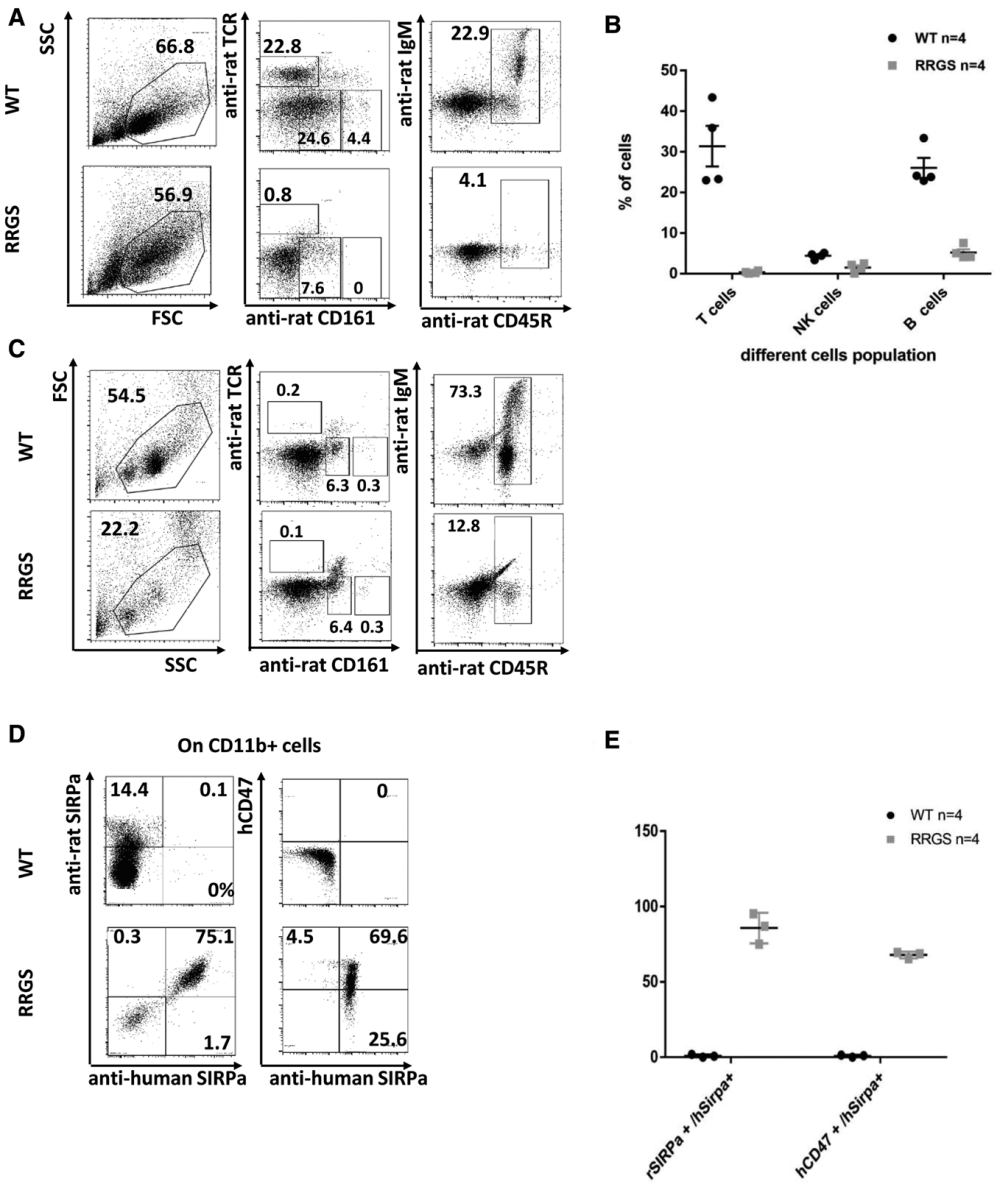
Cytometry analysis of rat leukocytes and hSIRPa expression in RRGS animals. PBMCs from spleen and bone marrow were collected from WT and RRGS animals and stained with the indicated antibodies and with hCD47Fc. A, Representative dot plots of T, NK, and B cells in the spleen of one RRGS animal. Numbers within dot plots represent the percentage of positive cells. B, Mean proportion in the spleen of T, NK, and B cells ±SD of n = 4 RRGS animals analyzed. C, Representative dot plots of T, NK, and B cells in the bone marrow of 1 RRGS animal. Numbers within dot plots represent the percentage of positive cells. D, Representative dot plot rat CD11b+ cells labeled with antirat and antihuman SIRPa mAbs as well as with hCD47 and anti-hSIRPa in WT and RRGS animals. Numbers within dot plots represent the percentage of positive cells. E, Mean proportion among rat CD11b+ cells of rat SIRPa+ and human SIRPa+ cells as well as of hCD47+ and human SIRPa+ cells in 4 RRGS and 4 WT animals analyzed. FSC, forward scatter; NK, natural killer; PBMC, peripheral blood mononuclear cells; RRGS, Rat Rag1-deficient, Il2rg-deficient, hSIRPa+; SIRP, signal regulatory protein; SSC, side scatter; TCR, T cell receptor; WT, wild type.

Cytofluorimetric analysis of immune cells in the spleen (Figure 1A and B) and bone marrow (Figure 1C) of RRGS animals showed the absence of mature T, B, and NK cells. Identical phenotype was also already reported for RRG animals.^10^ LN were severely atrophic due to the absence of lymphoid development, as it is observed in immunodeficient mice^1,2^ and RRG animals^10^ and were not analyzed for cell composition.

A large majority of CD11b^+^ cells (among mononuclear cells only monocytes/macrophages due to the absence of NK cells) from spleen from RRGS were hSIRPa^+^ and all of these cells were also rat SIRPa^+^ (Figure 1D and E). Furthermore, hSIRPa^+^ monocytes/macrophages showed specific binding of human CD47, one of the ligands of SIRPa, whereas rat SIRPa+ macrophages from WT animals did not (Figure 1D and E).

Thus, RRGS have a profound immunodeficient phenotype for T, B, and NK compartments and all rat macrophages express a functional hSIRPa, in accordance with the genomic regulatory sequences of hSIRPa present in hSIRPa transgenic rats,^17^ that drive expression to myelomonocytic cells.^21^

### Immune Humanization Using PBMCs and aGVHD

In RRG animals, humanization with tissues or cells such as human skin, hepatocytes, or tumor cells was possible but preliminary experiments showed that immune humanization with PBMCs was not possible.^10^ To obtain immune humanization, RRG received different amounts of hPBMCs (from 230 to 1840 × 10^6^cells/kg) with or without irradiation and with no other treatment. These animals did not show detectable hPBMCs in blood and they did not display any sign of aGVHD (Table 1). As a comparison, NSG mice receiving 300 × 10^6^cells/kg hPBMCs develop aGVHD.^1,2^ To obtain preliminary information on the role of rat macrophages on the elimination of human cells, we treated RRG animals with liposomes containing clodronate which efficiently eliminates macrophages from organs with fenestrated endothelium, such as the spleen and liver.^20^

**TABLE 1.**
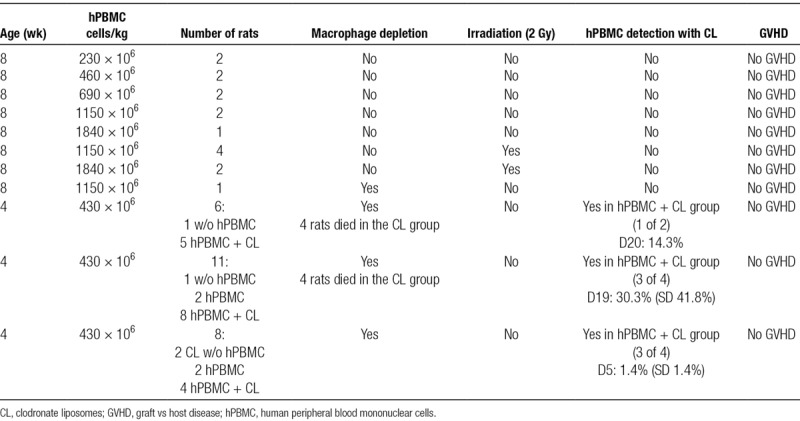


**FIGURE 2.**
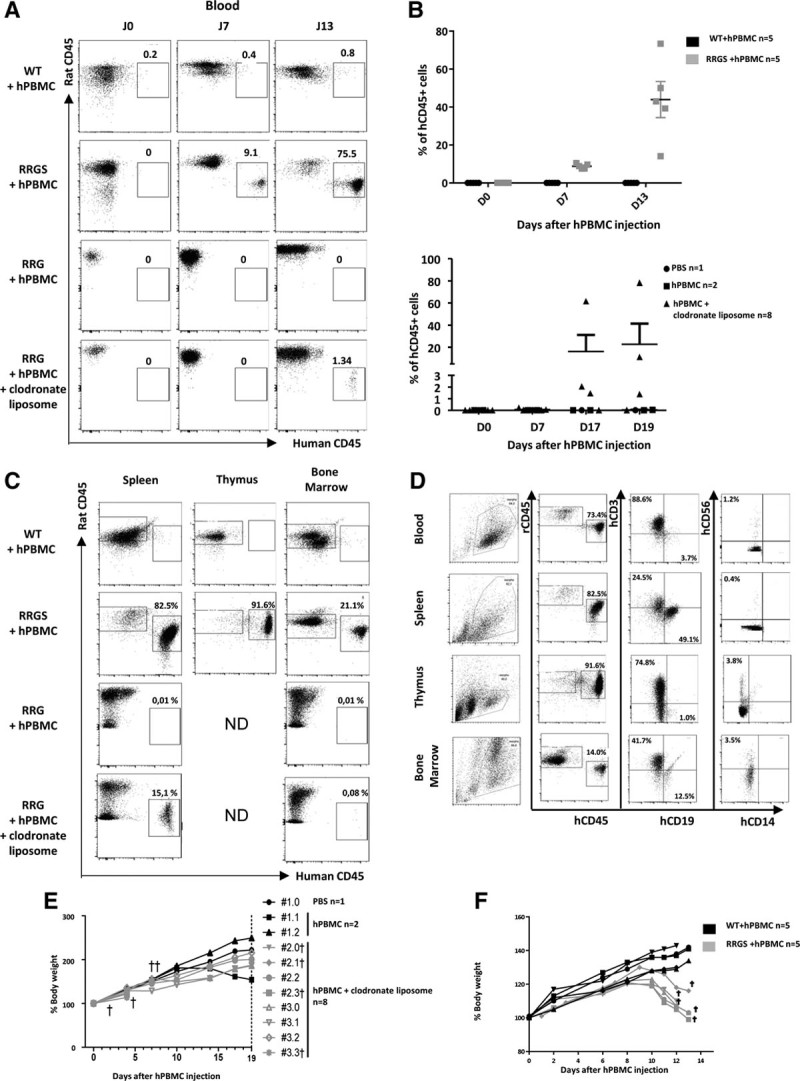
aGVHD in RRGS animals injected with hPBMCs. hPBMCs from healthy volunteers were injected iv (430 × 10^6^ cells/kg) in RRG, RRGS, or WT animals. RRG animals received or not treatment with clodronate liposomes. A, Representative dot plots of blood performed after hPBMC injection for detection of rat and human CD45^+^ cells. Numbers within dot plots represent the percentage of positive cells. B, Upper: Mean ± SD proportion of hCD45+ cells in blood of RRGS animals (n = 4). Lower: Mean ± SD proportion of hCD45+ cells in blood of RRG animals with (n = 8) or without (n = 2) clodronate liposomes. C, Representative dot plots of hCD45+ cells in spleen, thymus, and bone marrow at euthanization (d 17 and 19 after PBMC injection for RRGS and RRG, respectively). Numbers within dot plots represent the percentage of positive cells. D, Subset composition of hCD45^+^ cells in blood and spleen of an RRGS animal euthanized at day 17 after hPBMCs injection. CD3 for T cells, CD19 for B cells, CD56 for NK cells, and CD14 for monocytes. E, Weight curves of RRG animals injected or not with hPBMCs and with or without clodronate liposomes. †Animals that died spontaneously without any signs of aGVHD. F, Weight curves of RRGS animals injected or not with hPBMCs. †Sacrificed animals when weight loss and other clinical signs showed irreversible aGVHD. aGVHD, acute graft vs host disease; hPBMC, human peripheral blood mononuclear cells; iv, intravenously; ND, not done; NK, natural killer; PBS, phosphate buffer solution; RRG, Rat Rag1-deficient, Il2rg-deficient; RRGS, Rat Rag1-deficient, Il2rg-deficient, hSIRPa+; WT, wild type.

When RRG animals received hPBMCs (430 × 10^6^ cells/kg) and were treated with liposomes containing clodronate and thereafter twice a week for 19 days, they showed variable levels (0%, 1.3%, 5%, and 95%, n = 4) of hCD45^+^ leukocytes in blood and spleen and in 1 of 3 animals in the bone marrow (Figure 2A–C). RRG animals treated with clodronate liposomes showed early toxicity (4 of 8 died before day 7 without weight loss or any other sign of aGVHD) (Figure 2E and Table 1) and we did not irradiate them to avoid further toxicity. Those with hCD45+ cells (animals 3.0, 3.1, and 3.2) did not show signs of aGVHD and showed normal weight curves (Figure 2E). After the withdrawal of clodronate liposome treatment during 5 weeks, leukocyte humanization was progressively lost over the 2 following weeks (from 90% to 12%, respectively, data not shown).

All these data supported the hypothesis that immune humanization was precluded by rat macrophages.

Human SIRPa expression by rat macrophages should inhibit the phagocytosis of CD47-positive human cells by rat macrophages through delivery of “don’t eat me signals.” hPBMCs (430 × 10^6^ cells/kg) injected into RRGS animals resulted in a rapid and reproducible detection of human CD45^+^ lymphocytes in blood, spleen, thymus, and bone marrow (Figure 2A–C). Human CD45^+^ leukocytes in blood were detectable at day 7 and increased by day 13. The proportion of hCD45+ cells was very high in spleen and thymus and lower in the bone marrow (**Figure S1, SDC**, http://links.lww.com/TP/B843). The number of hCD45+ cells in spleens at sacrifice was 95.5 ± 58 × 10^6^ cells (n = 4). hCD45+ cells in blood and spleen were composed by a high proportion of T and B cells, a small fraction of NK cells and rarely of monocytes (Figure 2D). T cells were composed of both CD4+ and CD8+ cells in a 2:1 ratio (data not shown). Within the first 2 weeks following hPBMC injection, all RRGS consistently developed an aGVHD as shown by weight loss (Figure 2F) as well as other clinical signs, such as hutching, diarrhea, skin lesions, abnormal fur, and reduction of locomotion (**Figure S2, SDC**, http://links.lww.com/TP/B843).

Alanine and mainly aspartate transaminases were elevated at 7 days and much higher at 13 days after hPBMC injection indicating hepatocyte destruction (**Figure S3, SDC**, http://links.lww.com/TP/B843).

Injection of lower number of hPBMCs, 215 × 10^6^ or 161 × 10^6^ cells/kg, also resulted in aGVHD with slower kinetics and only in a small fraction of the animals when using the lowest dose in the tumor model (data not shown and **Figure S5, SDC**, http://links.lww.com/TP/B843, respectively).

In summary, injection of hPBMC resulted in a rapid, robust, and reproducible immune humanization mainly composed of T and B cells and as a consequence in a dose-dependent aGVHD and fatal GVHD.

### aGVHD in RRGS Animals as a Model to Apply New Treatments: Use of LIS1, a New Porcine Antihuman Lymphocyte Antibody

**FIGURE 3.**
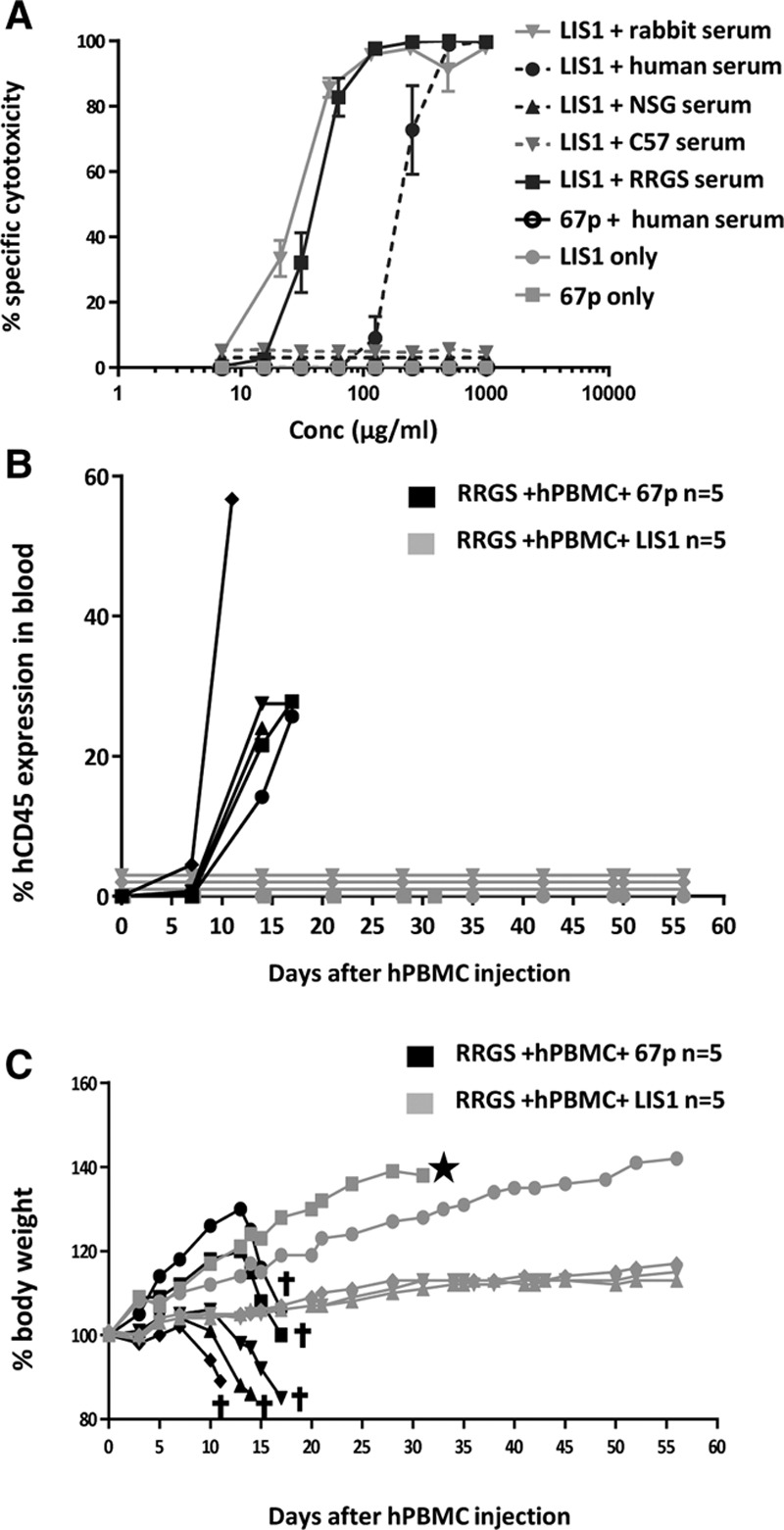
Evaluation of a new antilymphocyte antibody (LIS1) for the treatment of aGVHD in RRGS animals. A, Evaluation of CDC using hPBMCs was performed by in vitro incubation with pure rat, human, NSG, C57/Bl6 serum as a source of complement and increasing doses of purified pig LS1 or control nonimmune (67p) IgG (n = 3 donors for each serum species used in 3 independent experiments). B, hPBMCs from healthy volunteers were injected iv (215 × 10^6^ cells/kg) to RRGS animals treated with LIS1 or control IgG (67p) injected from the day of hPBMC injection biweekly until d 28. Proportion of hCD45^+^ cells in blood at different time points on LIS1 or control IgG (67p)-treated RRGS. C, Weight curves of LIS1 or control IgG (67p)-treated RRGS. †Euthanized animals due to >20% weight loss. *Euthanized animal for analysis of hCD45^+^ cells. aGVHD, acute graft vs host disease; CDC, complement-dependent cytotoxicity; hPBMC, human peripheral blood mononuclear cells; iv, intravenously; LIS1, pig purified IgG antihuman lymphocytes; NSG, NOD scid gamma; RRGS, Rat Rag1-deficient, Il2rg-deficient, hSIRPa+.

We aimed to test a new complement-activating pig antihuman lymphocyte antibody on this RRGS model of aGVHD. To this end, we first tested in vitro the CDC capacity of the LIS1 antibody using as a source of complement rat, rabbit, or human sera (Figure 3A). As comparative controls, we used mouse (NSG and C57Bl/6) sera because immunodeficient variants from these strains are the most commonly used in aGVHD models. LIS1 antibody induced the most potent CDC in the presence of rat or rabbit sera (50% CDC at ~30 µg/mL of LS1) followed by human sera (50% lysis at ~150 µg/mL). CDC was undetectable in the presence of NSG or C57Bl/6 mouse complement emphasizing the interest of RRGS versus NSG animals (Figure 3A). RRGS animals were then injected with hPBMCs and with LIS1 or control pig IgG (67p) at 40 mg/kg from day 0 and biweekly until day 28. The animals were followed for hCD45^+^ cells in blood and clinical signs of aGVHD. All antibody control-treated RRGS animals showed rapid appearance of hCD45^+^ cells in blood with high levels (20%–50%) at euthanization when they reached >20% weight loss. In contrast, none of the LIS1-treated RRGS animals had detectable hCD45R^+^ cells in the blood neither at early nor at late (day 57) time points after treatment withdrawal (Figure 3B). One LIS1-treated RRGS animal euthanized at day 30 did not show detectable hCD45+ cells neither in the blood not in the spleen (data not shown). All antibody control-treated RRGS animals developed fatal aGVHD as shown by >20% weight loss before day 17 (Figure 3C) as well as all the other clinical signs (Fig**ure** S4, **SDC**, http://links.lww.com/TP/B843) whereas all LIS1-treated RRGS animals gained weight (Figure 3C) and did not have other clinical signs of aGVHD (Fig**ure** S4**, SDC**, http://links.lww.com/TP/B843) at least until day 60.

Thus, RRGS constitute a useful model for the study of complement-activating depleting antibodies and this new antilymphocyte antibody is shown for the first time to be a very effective treatment of human immune responses in vivo, particularly for aGVHD.

### RRGS Animals as a Model of Cellular Human Antitumor Immune Responses

**FIGURE 4.**
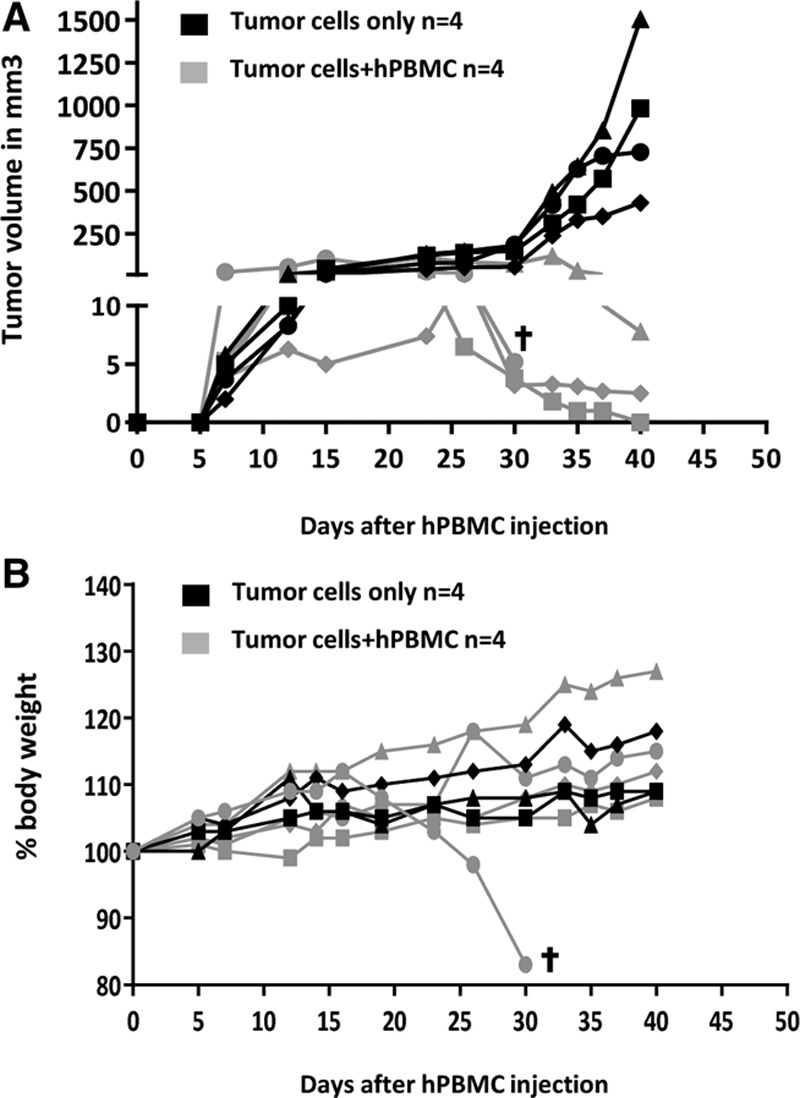
Tumor model and antitumor immune response using hPBMCs. Human mammary tumor cells (B2 cell line) were injected subcutaneously in RRGS animals (n = 8) and when tumors were measurable (d 0), RRGS animals were injected iv with hPBMCs (161 × 10^6^ cells/kg, n = 4) or PBS (n = 4). A, Tumor volume was determined at the indicated time points in RRGS animals injected or not with hPBMCs. B, Weight curves of RRGS animals injected or not with hPBMCs. †Euthanized animal due to weight loss >20%. hPBMC, human peripheral blood mononuclear cells; iv, intravenously; PBS, phosphate buffer solution; RRGS, Rat Rag1-deficient, Il2rg-deficient, hSIRPa+.

To assess human immune responses against tumors in RRGS, we implanted subcutaneously a human mammal cancer cell line, and when tumors were detectable (day 7), we injected hPBMCs at a dose that would not induce a rapid aGVHD (161 × 10^6^ cells) and analyzed both the growth of the tumors and signs of aGVHD (Figure 4). Tumor growth was inhibited in all RRGS animals only when injected with hPBMCs (Figure 4A). The weight curve of RRGS animals injected or not with tumors increased with the exception of 1 RRGS animal treated with PBMCs that died of aGVHD at day 30 but without detectable tumor (Figure 4B). This animal had a high level of hCD45R^+^ cells in blood (45.8%), whereas the other animals showed low or no signs of aGVHD and survived (Fig**ure** S5, **SDC**, http://links.lww.com/TP/B843).

## DISCUSSION

Although immune humanized mouse models are very useful in a wide variety of models, immune humanization of immunodeficient rats have advantages in certain aspects. Rats are 10 times bigger than mice and analysis of certain models may beneficiate of this larger size, such as implantation of human tumors in small anatomical locations such as prostate and precise locations in the brain or of organoids in orthotropic locations. Inbred mouse strains have very low complement activity whereas rats have complement levels comparable to humans, as previously shown for several strains of each species^3^ and in this work specifically comparing NSG and RRGS serum to obtain cytotoxicity using a new complement-activating antilymphocyte antibody. Another advantage of rats is that following injection of hPBMCs and aGVHD, the number of human cells obtained from a spleen of RRGS animals (50–150 × 10^6^ cells) is much higher than those obtained from spleens of immunodeficient mice with an aGVHD (2–10 × 10^6^ cells; data not shown). Therefore, functional and molecular studies in subpopulations of human cells, such as Treg, during activation, and aGVHD, are more feasible using RRGS rather than NSG animals.

A weakness of human aGVHD models in immunodeficient mice is the use of total body irradiation to observe clear clinical GVHD, which is increasingly disparate to clinical practice, and which is not needed in this RRGS model. In addition, *Prkdc* mutations to obtain a severe combined immunodeficient phenotype in many mouse strains (like in all NOD-derived immunodeficient strains such as NSG and NOG) and several rat immunodeficient models^6,16^ limit the use of irradiation needed in certain models such as in cancer treatments because PRKDC is an enzyme essential in DNA nonhomologous end joining and this generates uncontrolled toxicity in the host tissues. RRGS animals do not have mutations in the *Prkdc* gene and should be thus more adapted to this kind of experiment.

A series of rat immunodeficient models are available. The first of these rats and the only for many years were nude rats due to a mutation in the *Foxn1* gene.^22^ Nevertheless, nude rats as nude mice are only T-cell deficient, while B and NK cells are normal. Furthermore, they have a leaky phenotype that makes that older animals have T cells.^23,24^ In more recent years, a series of immunodeficient rats due to mutations in other genes have been generated, including animals deficient for Rag1,^7,8^ *Rag2*,^25^ or *Il2rg.*^26^ Nevertheless, the *Rag1* and *Rag2* KO rats have normal NK cells and there is residual B and T cells for *Rag1*, *Rag2*, or *Il2rg* mutated animals. More severe immunodeficient animals combining several mutations of the above-mentioned genes have been more recently described.^6,8-10^ A very recent publication with combined mutations for *Prkdc*, *Il2rg*, and expression of the hSIRPa allowed better immune humanization compared with animals without hSIRPa.^16^ Our results confirm the beneficial effect of hSIRPa to obtain immune humanization and extend for the first time the use of these animals to models of aGVHD and antitumor immune responses.

In the RRGS aGVHD model, we confirmed the potential of a new antihuman T-cell polyclonal antibody functioning through complement activation to be applied in human GVHD. The levels of complement in the large majority of inbred mouse inbred strains,^3^ including NOD-derived immunodeficient animals,^14,15^ is undetectable or very low. This led to the recent development of a complement-sufficient NSG strain.^27^ Nevertheless, NOD mice also lack NK cells^15^ and are thus not suitable for the analysis of ADCC mechanisms. Because LS1 antibody does not kill directly human cells in the absence of complement, the complete elimination in vivo was due to CDC and/or ADCC in proportions that need to be analyzed in the future.

Our results also show for the first time human antitumor immune responses allowing in the future to use RRGS animals in experiments aiming to evaluate these responses.

Immune humanization of RRGS using human CD34+ hematopoietic precursors will be the objective of new studies. Previous study with another rat line showed that this is feasible when using at the same time hCD34+ cells from fetal liver and fetal thymus but the degree of humanization was low as compared with NSG mice.^16^

The use of immunodeficient rats has limitations compared with immunodeficient mice because their larger size implies the use of a larger space in animal facilities and thus a higher cost. Also, their larger size demands the use per body weight of larger amounts of cells or molecules to obtain the same effect.

In conclusion, RRGS animals were efficiently immune humanized using PBMCs and human aGVHD and antitumor immune responses could be detected. Furthermore, a new antilymphocyte antibody was used for the first time to inhibit in vivo human immune responses.

## ACKNOWLEDGMENTS

This work was also realized in the context of the support provided by the UPGRADE H2020 Consortium (Grant agreement no. 825825) and by the ReSHAPE EU Horizon 2020 (Grant agreement no. 825392).

## Supplementary Material


