# Supplementary material for: In Vivo Analysis of Human Immune Responses in Immunodeficient Rats
Source: Transplantation. 2020 Mar 31;104(4):715–23. doi: 10.1097/TP.0000000000003047 (PMC7147402; doi:10.1097/TP.0000000000003047)
Supplement: Supplementary file 1 [file tp-104-715-s001.pdf]

Figure S1.

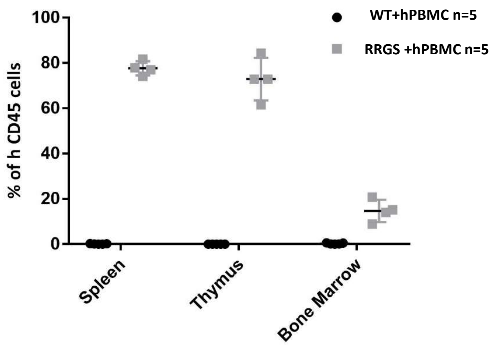

Figure S1: Analysis of the proportion of hCD45+ cells in RRGS animals immune humanized with hPBMCs. RRGS or WT animals (n=5 each) were injected iv with hPBMCs (430.10^6 cells/Kg) from healthy volunteers and sacrificed at day 13 after hPBMCs infusion. hCD45+ cells were analyzed in the indicated organs.

Figure S2.

		hutching	fur	skin	activity	diarrhea	weight loss	total
	**1**	0	0	0	0	0	0	**0**
**WT+hPBMC**	**2**	0	0	0	0	0	0	**0**
**n=5**	**3**	0	0	0	0	0	0	**0**
	**4**	0	0	0	0	0	0	**0**
	**5**	0	0	0	0	0	0	**0**
	**1**	1 D11	1 D11	1 D12	1 D11	0	1 D11	**5**
**RRGS+hPBMC**	**2**	1 D11	1 D11	1 D12	1 D11	0	1 D11	**5**
**n=5**	**3**	1 D11	1 D11	1 D12	1 D11	0	1 D12	**5**
	**4**	1 D10	1 D11	1 D11	1 D10	0	1 D11	**5**
	**5**	1 D10	1 D11	1 D11	1 D10	0	1 D11	**5**

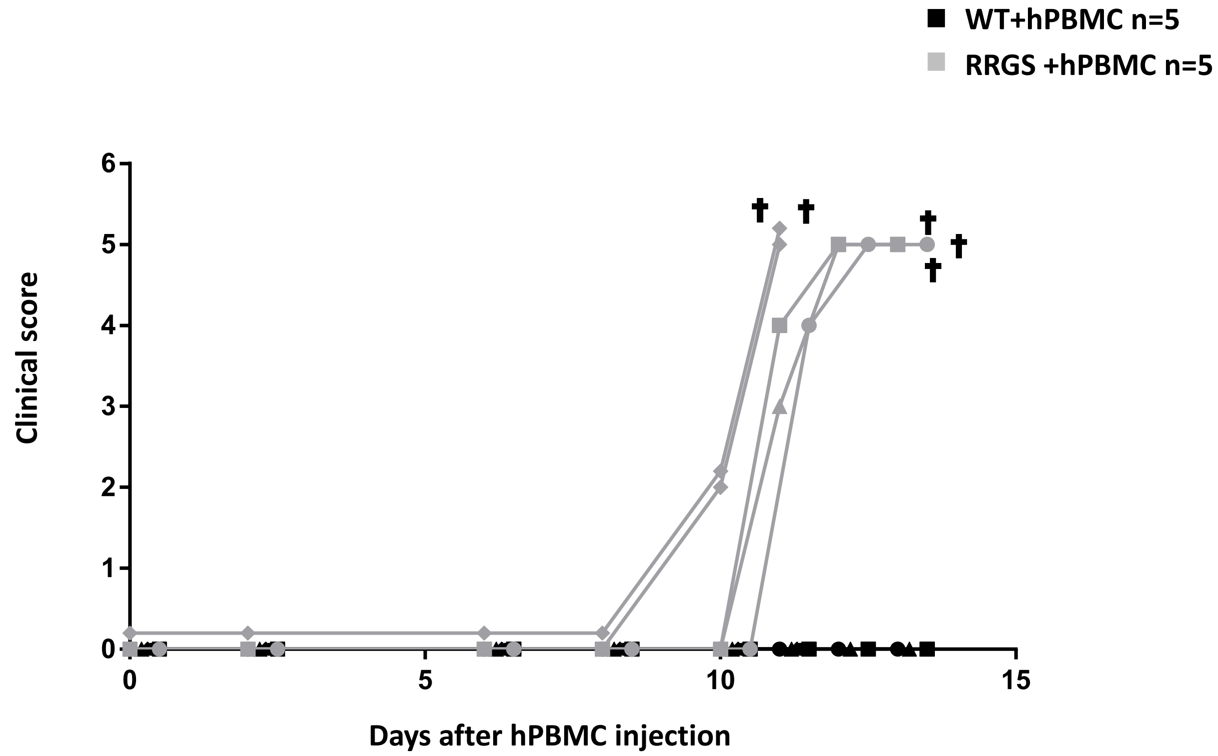

Figure S2. aGVHD clinical score of RRGS animals injected with hPBMCs. hPBMCs (430.10^6 cells/Kg) from healthy volunteers were injected iv in RRGS or WT animals. Different clinical parameters; hutching, rough fur, skin lesions, decreased physical activity, diarrhea, and weight loss were scored as 0=no signs, 1=present. WT+hPBMCs, n=5. RRGS+hPBMCs, n=5.

Figure S3

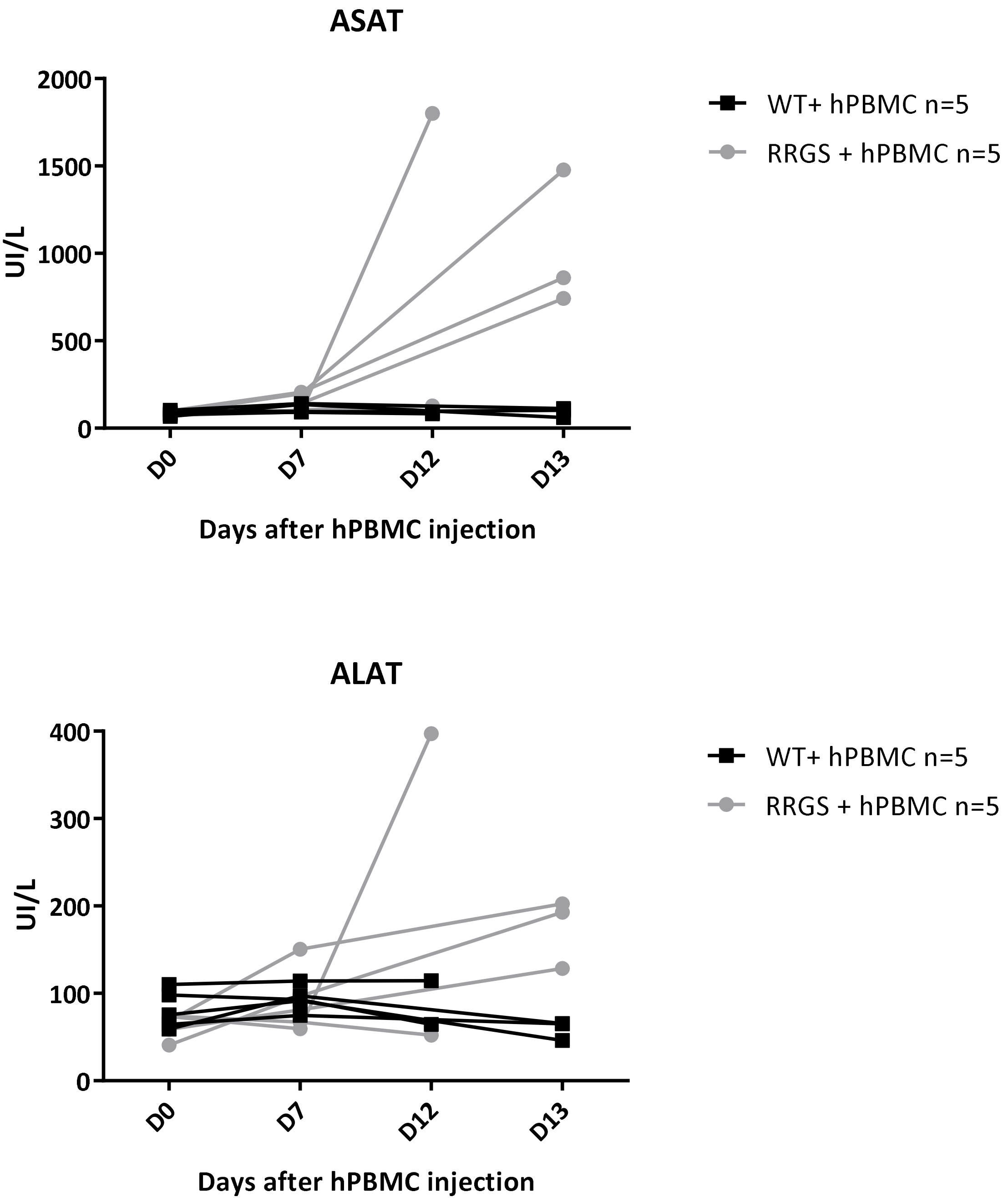

Figure S3. Alanine and aspartate transaminases (ALT and AST) in serum during aGVHD in RRGS animals. hPBMCs (430.10^6 cells/Kg) from healthy volunteers were injected iv in RRGS or WT animals and serum was harvested at the indicated time points for analysis of AST and ALT. WT+hPBMCs, n=5. RRGS+hPBMCs, n=5.

Figure S4

		hutching	fur	skin	activity	diarrhea	weight loss	total
**RRGS+ hPBMC+ 67p n=5**	**1**	1 D15	1 D15	1 D17	1 D15	0	1 D14	**5**
	**2**	1 D15	1 D15	1 D17	1 D15	0	1 D14	**5**
	**3**	1 D13	1 D13	0	1 D13	0	1 D10	**4**
	**4**	1 D11	1 D11	1 D11	1 D11	0	1 D10	**5**
	**5**	1 D15	1 D15	0	1 D15	0	1 D13	**4**
**RRGS+ hPBMC+ LIS1 n=5**	**1**	0	0	0	0	0	0	**0**
	**2**	0	0	0	0	0	0	**0**
	**3**	0	0	0	0	0	0	**0**
	**4**	0	0	0	0	0	0	**0**
	**5**	0	0	0	0	0	0	**0**

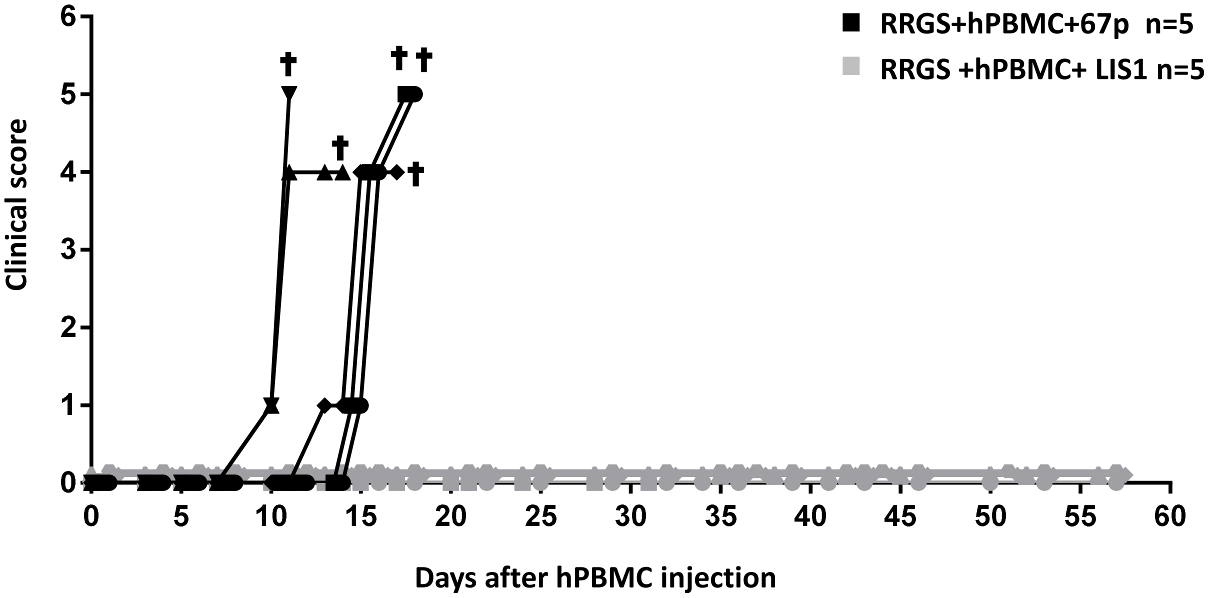

Figure S4. aGVHD clinical score of RRGS animals injected with hPBMCs treated or not with LIS1. hPBMCs from healthy volunteers were injected iv (215.10^6 cells/Kg) in RRGS animals treated or not with LIS1 or 67p (antibody control). Different clinical parameters; hutching, rough fur, skin lesions, decreased physical activity, diarrhea, and weight loss were scored as 0=no signs, 1=present. RRGS+hPBMCs+67p, n=5. RRGS+hPBMCs+LIS1, n=5.

Figure S5

		hutching	fur	skin	activity	diarrhea	weight loss	total
Tumor cells+ hPBMC n=4	**1**	0	0	0	0	0	0	**0**
	**2**	0	0	0	0	0	0	**0**
	**3**	0	0	0	0	0	0	**0**
	**4**	0	0	0	0	0	0	**0**
Tumor cells only n=4	**1**	1 D26	1 D23	0	1 D23	0	1 D19	**4**
	**2**	0	0	0	0	0	0	**0**
	**3**	0	0	0	0	0	0	**0**
	**4**	0	0	0	0	0	0	**0**

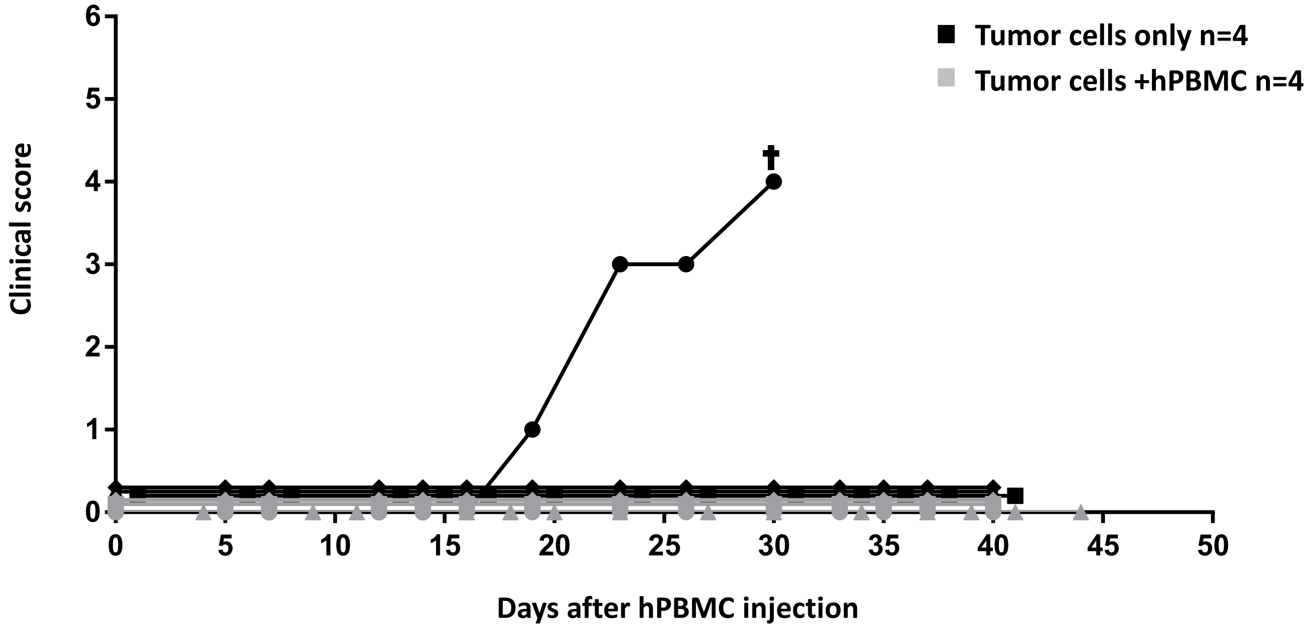

Figure S5. aGVHD clinical score of RRGS animals injected with hPBMCs and tumors. RRGS animals were injected with a human tumor cell line (B2 cells line) and then injected or not with hPBMCs iv (161.10^{6} cells/Kg). Different clinical parameters; hutching, rough fur, skin lesions, decreased physical activity, diarrhea, and weight loss were scored as 0=no signs, 1=present. RRGS+ tumor cells, n=4. RRGS+ tumor cells + hPBMC, n=4. One out of 4 RRGS+hPBMC animals developed aGVHD.
